# Do Ty3/Gypsy Transposable Elements Play Preferential Roles in Sex Chromosome Differentiation?

**DOI:** 10.3390/life12040522

**Published:** 2022-04-01

**Authors:** Kornsorn Srikulnath, Syed Farhan Ahmad, Worapong Singchat, Thitipong Panthum

**Affiliations:** 1 Animal Genomics and Bioresource Research Unit (AGB Research Unit), Faculty of Science, Kasetsart University, 50 Ngamwongwan, Chatuchak, Bangkok 10900, Thailand; syedfarhan.a@ku.th (S.F.A.); worapong.singc@ku.ac.th (W.S.); thitipong.pa@ku.th (T.P.); 2 Laboratory of Animal Cytogenetics and Comparative Genomics (ACCG), Department of Genetics, Faculty of Science, Kasetsart University, 50 Ngamwongwan, Chatuchak, Bangkok 10900, Thailand; 3 Special Research Unit for Wildlife Genomics (SRUWG), Department of Forest Biology, Faculty of Forestry, Kasetsart University, 50 Ngamwongwan, Chatuchak, Bangkok 10900, Thailand; 4 The International Undergraduate Program in Bioscience and Technology, Faculty of Science, Kasetsart University, 50 Ngamwongwan, Chatuchak, Bangkok 10900, Thailand; 5 Interdisciplinary Graduate Program in Bioscience, Faculty of Science, Kasetsart University, 50 Ngamwongwan, Chatuchak, Bangkok 10900, Thailand; 6 Amphibian Research Center, Hiroshima University, 1-3-1 Kagamiyama, Higashihiroshima 739-8526, Japan

**Keywords:** sex chromosome, sex determination, suppression recombination, transposable element, Ty3/Gypsy

## Abstract

Transposable elements (TEs) comprise a substantial portion of eukaryotic genomes. They have the unique ability to integrate into new locations and serve as the main source of genomic novelties by mediating chromosomal rearrangements and regulating portions of functional genes. Recent studies have revealed that TEs are abundant in sex chromosomes. In this review, we propose evolutionary relationships between specific TEs, such as Ty3/Gypsy, and sex chromosomes in different lineages based on the hypothesis that these elements contributed to sex chromosome differentiation processes. We highlight how TEs can drive the dynamics of sex-determining regions via suppression recombination under a selective force to affect the organization and structural evolution of sex chromosomes. The abundance of TEs in the sex-determining regions originates from TE-poor genomic regions, suggesting a link between TE accumulation and the emergence of the sex-determining regions. TEs are generally considered to be a hallmark of chromosome degeneration. Finally, we outline recent approaches to identify TEs and study their sex-related roles and effects in the differentiation and evolution of sex chromosomes.

## 1. Introduction

Sex determination is the process by which organisms develop as either male or female. Sex determination mechanisms differ among species. Under environmental sex determination, sexes do not differ in genotype but are determined by temperature changes, social factors, size, age or the presence of parasites [1,2,3], whereas under genotypic sex determination, sex is determined by genetic elements, mainly sex determining (SD) genes present in homomorphic to highly differentiated heteromorphic sex chromosomes (male heterogametic (XX/XY) or female heterogametic (ZZ/ZW)) [4,5,6,7]. In mammals, the male master gene (*SRY*), located on the Y chromosome, encodes functions involving testis development and is, therefore, the main player causing sex determination [8,9]. In medaka fish (*Oryzias latipes*, Temminck and Schlegel, 1846) [10], the Y-linked master gene *dmrt1bY*, which is a Y-specific duplicate of the *dmrt1* gene, drives development toward the male phenotype [11,12].

Recombination suppression is a critical step in sex chromosome evolution, in addition to the emergence of SD genes, the accumulation of repetitive sequences, the degeneration of W/Y sex chromosomes and, perhaps, the dosage compensation effect of Z/X sex chromosomes [4,13,14,15,16,17,18,19,20,21]. Recombination suppression prevents the transfer of SD genes to other chromosomes and thus preserves the integrity of the emerging sex chromosomes, rendering the Y or W chromosomes different from their X or Z counterparts [22]. A high-resolution genetic linkage map of papaya (*Carica papaya*, Linnaeus, 1753) [23], which has homomorphic sex chromosomes, revealed that 225 out of 347 markers co-segregated with sex phenotype, suggesting extreme recombination suppression around the sex-determining region (SDR) [24]. This has been also observed in spinach (*Spinacia oleracea*, Linnaeus, 1753) [25], which is a diploid dioecious leafy vegetable with a pair of homomorphic sex chromosomes [26,27,28,29]. The size of the recombination suppression region and differences in size between the X and Y or Z and W chromosomes may reflect the stage of sex chromosome evolution [6,16,30]. It has been postulated that most sex chromosomes in mammals and avians are ancient, whereas most homomorphic sex chromosomes in plants, insects, fishes, amphibians and reptiles have evolved recently [31,32,33,34]. The accumulation of deleterious mutations in most functional genes and expansion of repetitive DNA in the suppression recombination region result in sex chromosome degeneration [13,35,36,37,38,39].

The process of sex chromosome degeneration and rearrangement, in which an obvious increase in repetitive non-coding sequences and transposable elements (TEs) is observed for most W/Y chromosomes [17,18,19,20,21,40,41,42,43,44], is an important biological constraint that sheds light on the evolution of sex chromosomes [35,42,45,46]. TEs may play a significant role in sex chromosome differentiation by allowing W/Y chromosomes to achieve a state of beneficial non-homology/non-recombination via TE insertions in a short time [22,35,42,47,48]. The abundance of transposable elements in Z/X and W/Y chromosomes also suggests the emergence of SDRs [49,50]. These loci were enriched with highly sex-specific sequences and were formed from massive contents of TEs that became silenced and heterochromatic [51].

**Figure 1:**
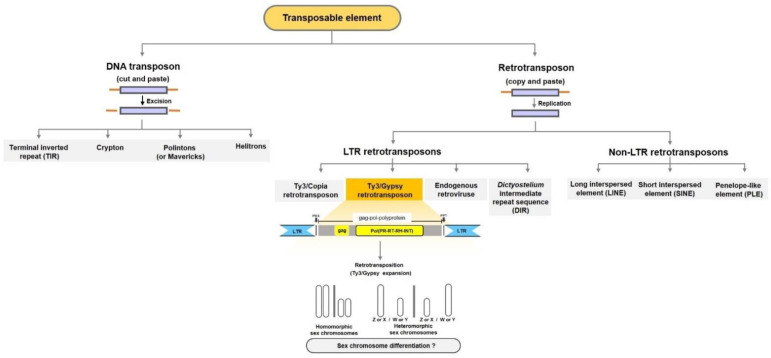
An overview of TE classification and their possible role in sex chromosome differentiation. The structure of Ty3/Gypsy TEs is highlighted, and a hypothesis regarding their possible role in sex chromosome differentiation is proposed as long terminal repeat: LTR; primer binding site: PBS; polypurine tract: PPT; group specific antigen: gag.

TEs are DNA segments that can move from one genome location to another. They are categorized into two main classes: (I) retrotransposons, which move by reverse transcription of an RNA intermediate via the “copy and paste” mechanism, and (II) DNA transposons, which move by a more straightforward mechanism of excision and insertion, known as the “cut and paste” mechanism [52,53,54]. All retrotransposons encode reverse transcriptase, except for non-autonomous elements that use enzymes of autonomous elements to retrotranspose into other genomic regions. Retrotransposons are further classified according to their structure and mechanism of transposition. Five major orders are distinguished: long terminal repeats (LTRs, including LTR retrotransposons and retroviruses), *Dictyostelium* intermediate repeat sequence (DIRS) and long interspersed elements (LINEs), which are autonomous partners of short interspersed elements (SINEs) and Penelope. DNA transposons use various enzymes, such as transposase, and are classified as terminal inverted repeats (TIRs), which encode a transposase, Crypton, which uses a tyrosine recombinase, Polintons (or Mavericks), which are self-synthesizing elements encoding up to 10 proteins, and Helitrons, which replicate via a rolling-circle mechanism using recombinase and helicase [55] (Figure 1).

As mobile elements, TEs can replicate and spread within genomes through transposition, leading to an increase in copy number by intrinsic replication, as in the case of retrotransposons, or the repair of double-strand breaks generated during transposition [56]. The insertion of TEs has been postulated to be one of the earliest triggers causing recombination suppression [57,58]. Some TEs are unequally distributed in sex chromosomes. In the sex chromosomes of papaya, the hermaphrodite-specific region in the Y chromosome (HSY) is highly abundant in interspersed repeats, including Ty1/Copia and Ty3/Gypsy, compared to the corresponding X chromosome and autosomes [59,60,61,62]. The Y chromosome of spinach contains a large number of Ty1/Copia -like derivative elements around the male-determining locus, whereas both Y sex chromosomes of the XY_1_Y_2_ system in sorrel (*Rumex acetosa*, Linnaeus, 1753) [63] harbor numerous Ty3/Gypsy [29,64]. The insertion of TEs into the regulatory region of *dmrt1bY* on the medaka (*O. latipes*) sex chromosome rewired the gene regulatory network cascades of sex determination [65]. Whether plant and animal sex chromosomes preferentially accumulate specific TEs compared to the other chromosomes remains unclear.

TEs can be tethered with specific chromatin proteins involved in recombination, and TEs may be subject to sex-specific regulation. Interestingly, recent genome-wide comparative SNP studies of male and female individuals of several fishes and reptiles (snakeskin gourami (*Trichopodus pectoralis*, Regan, 1910) [66], North African catfish (*Clarias gariepinus*, Burchell, 1822) [67], bighead catfish (*Clarias macrocephalus*, Günther, 1864) [68], jade perch (*Scortum barcoo*, McCulloch and Waite, 1917) [69], Siamese cobra (*Naja kaouthia*, Lesson, 1831) [70] and green iguana (*Iguana*, Linnaeus, 1758) [71]) have revealed numerous sex-specific or sex-linked loci with high similarity to Ty3/Gypsy [72,73,74,75,76,77,78]. This suggests that the non-random distribution of Ty3/Gypsy in the genome may drive sex chromosome differentiation. Here, we discuss the evidence pertaining to different Ty3/Gypsy and other TE profiles obtained from comparative genomic research and propose a hypothesis that presents a comparative overview of TE landscapes in different species. The dynamics of Ty3/Gypsy and other TE-mediated sex chromosome modifications and their evolutionary impact on genome reorganization are also discussed.

## 2. Roles of TEs during Sex Chromosome Differentiation

Using comparative genomic tools, the dynamics of functional TEs on sex chromosomes can be identified in two aspects: (1) sequence conservation and (2) co-localization with regions with a known genomic function [79,80]. The conservation of TE sequences likely passively contributes to sex chromosome degeneration [81]. TE accumulation on the W/Y chromosome may start from several insertions in the close vicinity of crucial heterogametic sex-linked genes/loci where TEs can escape removal from the population due to stochastic processes, such as methylation/heterochromatization, ectopic recombination between TEs, or size reduction on W/Y sex chromosomes [62,82,83,84].

In contrast to mammals, birds and many snakes, which have small and degenerate W/Y chromosomes, the heterogametic sex chromosome (W/Y) is substantially larger than the Z/X chromosome in many fishes, reptiles and amphibians, indicating that sex chromosomes in these groups are usually younger, with frequent turnover [20,44,85,86,87,88,89,90]. In the medaka fish Y chromosome, the male-specific SDR has undergone duplication after TE insertion into the proto-Y chromosome [91]. In platyfish (*Xiphophorus maculatus,* Günther, 1866) [92], a higher repeat content was found in the SDRs of the X (43% repetitive sequences with 36% TEs) and Y (49% repetitive sequences with 32% TEs) chromosomes than in the whole genome (23% repeats with 21% TEs). The X and Y SDRs have higher densities of repeats and TEs than the whole X chromosome [93,94]. This suggests that TEs occupy the whole Y SDR, but are more compartmentalized on the X SDR [95]. A similar phenomenon occurs in the W chromosome of the half-smooth tongue sole (*Cynoglossus semilaevis*, Günther, 1873) [96], which has a substantially higher TE content than the Z chromosome (Chen et al. 2014) [97]. This indicates that TEs are recently strongly active in the X and Y or Z and W SDRs, thus potentially increasing the size and divergence of the currently small non-pseudo-autosomal region [98,99]. In the genome of the African clawed frog (*Xenopus laevis*, Daudin 1802) [100], recombination between the W and Z sex chromosomes stopped recently, with a strong accumulation of TEs in W-specific regions [101]. Potentially, the accumulation of TEs interfered with chromosome pairing during prophase I of meiosis and suppressed recombination, leading to an increase in sex chromosome size during the early phase of their differentiation, whereas a size reduction occurred later in their evolution and thus the W/Y sex chromosomes became smaller [102,103,104,105,106].

The transposition of a pre-existing SD locus onto a new chromosome may lead to the emergence of a new sex chromosome [107]. TEs displace DNA and may promote the emergence of new SD loci [108,109,110]. Recent studies have shown that the master SD gene, *sdY*, is conserved in many species. However, it is not consistently located on the same linkage homology but seems to behave like a “jumping gene” [109,111,112]. Analysis of the boundaries of the moving region that harbors *sdY* revealed the presence of several TE sequences, based on which a mechanism involving TE-associated transduction has been proposed [113]. Similarly, in medaka, TEs co-localize with regions that have a known genomic function and play an active role in the evolution of sex chromosomes, and the integration of DNA via TEs contributed to the regulation of the newly emerged SD gene, *dmrt1bY*. The SD gene of medaka arose from a duplication event of the autosomal *dmrt1a* gene [11]. The two *dmrt1* genes exert their functions at different times during gonad development: *dmrt1bY* in the SD stage and *dmrt1a* in the differentiating and adult testes [114]. This phenomenon may be linked to a rapid turnover of the sex chromosomes in the lineage, e.g., the genus *Oncorhynchus* has six independent sex chromosome pairs that originated 6–8 million years ago (MYA) [112].

The variety of Y chromosomes probably results from TEs (TC1-like transposase and non-LTR retrotransposons) in the flanking regions of the SDRs, which can move throughout the genome [110]. The movement of male/female SDRs among autosomes may prevent sex chromosome degradation and deleterious TE accumulation [95]. Alternatively, TEs have been shown to play a role in heterochromatization of the W/Y chromosomes and dosage compensation mechanisms [97,115,116,117,118,119,120,121]

TEs may play a role in resolving dosage-related gene expression problems in several species by promoting the silencing and condensation of sex chromatin in X (or Z) chromosomes, known as “the hitchhiking effect of favorable mutations” [35]. Interspersed repeat elements such as L1s in humans and mice have been suggested to enhance the inactivation process, thus promoting the heterochromatin state [122]. X chromosome inactivation in mammals, also termed “lyonization”, is a dosage compensation process in which one of the two X chromosomes is inactivated in XX females during early embryogenesis, preventing gene overexpression in comparison to males, which have a single X chromosome [123]. In marsupials, such as opossums, the paternal X chromosome is inactivated, whereas in placental mammals, a random X chromosome is inactivated by a long non-coding RNA produced by *Xist* [124]. Interestingly, in opossums, L1s show equally frequent interruptions on the X chromosome and autosomes, whereas in humans, L1s are less frequently interrupted on the X chromosome than on the autosomes [125]. On the human X chromosome, L1-poor regions contain genes that escape X inactivation and are physically distant from *Xist*-silenced regions [126]. This suggests that L1s play a role in the spreading of X chromosome silencing by recruiting *Xist* RNAs, which play a general role in the regulation of X-gene expression. This hypothesis has been tested in the spiny rat (*Tokudaia osimensis*, Abe, 1934) [127], of which males and females are XO [128,129]. A similar high concentration of LINEs was observed on both male and female X chromosomes, whereas no dosage compensation by X inactivation is required. This suggests that LINEs are not required on the X chromosome [130]. It is unclear whether the unique TE distributions patterns on the X chromosome are the cause or consequence of inactivation (or both). One possibility is that L1 accumulation on the X chromosome may be only a by-product of reduced recombination. Further investigation of the role of LINEs in X chromosome inactivation in mammals and other organisms is necessary.

Recently, the TE *drbx1* was found to be inserted in an intron of the X-linked region encoding the SD gene *dmrt1* in Siamese fighting fish (*Betta splendens*, Regan, 1910 [131]) [132]. This structural change was associated with a shift in the epigenetic silencing of X-*dmrt1* during the critical sex determination stage. Similar mechanisms have been found in plants, e.g., in melon (*Cucumis melo* L.), and TE-induced methylation of the promoter of the transcription factor *CmWIP1* has been shown to suppress expression and thus realize sex determination [133].

Some TE families are differentially expressed in specific tissues or conditions between sexes [81]. In platyfish, an accumulation of *Texim* genes is observed on the Y chromosome [134]. These genes are physically associated with a Helitron, which may have spread the *Texim* sequences on Y, but not X. The higher density of TEs on Y may suggest that they regulate some key sexual developmental genes and, consequently, impact sexual development [81]. In the medaka genome, a *dmrt1*-binding element in the promoter of the SD gene *dmrt1bY* mediates downregulation through its own gene product and autosomal *dmrt1a*. This *dmrt1*-binding silencer was introduced in the *dmrt1bY* promoter through the insertion of a novel TE, termed Izanagi, which is present in multiple copies in the genome and acts as a transcription factor-binding site [65]. This event contributed to the transcriptional rewiring of the new SD gene that created evolutionary innovations [135,136]. A recent study assessed TE expression on sex chromosomes of *Drosophila melanogaster* (Meigen, 1830) [137] and found specific enrichment of expressed TEs on the Y chromosome that were depleted on the X chromosome [138]. This result suggested that high-level expression of Y-specific TEs was associated with the activation of spermatocyte-specific and Y chromosome-specific transcriptional pathways.

The discovery of sex determination in the domestic silk moth (*Bombyx mori*, Linnaeus, 1758 [139]) demonstrated the regulatory function of the W chromosome, where TEs are involved in physical and biochemical interactions with thousands of autosomal protein-coding genes [116,118,140]. In the house fly (*Musca domestica*, Linnaeus, 1758 [119]), approximately two-thirds of the Y-linked scaffolds contain sequence similarities with TEs (Meisel et al. 2017 [120]). Neo-X chromosome formation through a domesticated non-autonomous Helitron has been identified in *Drosophila miranda* (Dobzhansky, 1935 [121]), and its role in the expression of X-linked genes has been revealed [117]. By contrast, the formation of male-specific lethal binding sites contributes to the dosage compensation process [117]. In mammalian genomes, TEs are hypomethylated in females compared to males, implying that oocytes are more resilient to TE transposition than the male germline. This may be linked to the lifelong division and numerous cell divisions of spermatogonial cells in contrast to oocytes. More cell divisions may allow for many deleterious insertions in the male germline [141].

In addition to the effects of TEs on host gene expression, there exist genomic differences between males and females in terms of TE profiles that impact sexual development [81]. Subsequent cycles of W/Y chromosome degeneration and rejuvenation may differ in length of evolutionary time. During W/Y chromosome degeneration, the appearance of a large number of TE transcripts in a cell may be more or less sudden [95]. In species/populations with a fixed W/Y chromosome loss or with frequent turnover, the period of TE transcript increase may be too short to induce trans-generational and constant preparedness of the genome defense system for TE invasion. When genome defense is inefficient, W/Y chromosome degradation may proceed at a faster rate, and the cycle of chromosome rejuvenation may be shorter [95]. The W/Y chromosome may become a substantial source of functional TE transcripts in the cell and threaten whole-genome stability [142,143,144,145]. The high density of TEs on the W/Y chromosome may serve as a hallmark for heterochromatin marks, which result in different levels of chromatin repression in the rest of the genome and in differential gene expression between males and females [35,36]. TE accumulation has been observed on specific Y or W sex chromosomes and on the corresponding regions of the X and Z chromosomes [83,146]. It is well established that TEs are abundant and play roles in both heterogametic and homogametic sex chromosomes; however, the question remains as to whether TEs are passive or active drivers of sex chromosome differentiation and evolution.

## 3. Non-Random Distribution of TEs on Sex Chromosomes across Different Lineages

Sex chromosome dynamics are related to the presence of TEs [17,18,19,20,21,40,44,83,90]. To further the understanding of the role of TEs in sex chromosome differentiation, the distribution of TEs across sex chromosomes of several lineages has been investigated [147,148]. TE profiles on the W chromosome of *Apareiodon* spp. (Eigenmann, 1916 [149]) were obtained via RepeatMasking and RepeatExplorer analyses of their genome sequences [104]. Three transposons—Helitron, Tc1-mariner, and EnSpm—showed major coverage in female genome clusters detected by RepeatExplorer and were accumulated in repeated copies in the W chromosome between 20 and 12 MYA [104]. By contrast, other TEs, such as DNA/Hat and Ty3/Gypsy, expanded in the genome before 20 MYA or after 12 MYA, with no consistent signals on sex chromosomes. This suggests that Helitron, Tc1-mariner and EnSpm are preferentially accumulated on the W chromosome in *Apareiodon* spp. and may play crucial roles in sex chromosome differentiation. Helitron, which is present in high copy numbers in the proximal region of the Z short arm and in the W long arm, may have caused recombination suppression between the Z and W chromosomes 20 MYA [22], followed by an accumulation of Tc1-mariner and EnSpm, which also exhibit a high copy number on the Z chromosome [104]. These elements can transform chromatin into a condensed form.

Genome sequences of the Chinese half-smooth tongue sole have provided a unique opportunity to compare TE profiles of full sequences between the Z and W chromosomes and autosomes (chromosomes 3 and 13) [97]. The TE content is substantially high on the W sex chromosome, followed by the Z sex chromosome. The high number of LINEs on the W chromosome concurs with the absence of recombination over nearly the entire length of the W chromosome [97]. Both the W and Z chromosomes underwent a recent accumulation of LINEs, while a second recent large wave of TE accumulation was observed in the region at approximately 7.5% of the W chromosome. This suggests that TE activity on the Z chromosomes decreased recently, whereas on the W chromosome, TEs may still be active [84]. The W chromosome also exhibits an abundance of endogenous retroviruses (ERVs), with 23 long copies of a single ERV element, whereas the other chromosomes do not harbor any full-length copies. A weak similarity with an exonuclease-endonuclease-phosphatase domain from some LINEs was also found, suggesting that a specific unclassified—and probably non-autonomous—element, potentially derived from TEs, may accumulate preferentially on the W chromosome of the Chinese half-smooth tongue sole [104]. A similar preferential accumulation of Tc1/mariner and L1 in non-recombining heterochromatic chromosomes has been documented on the Y long arm of the Antarctic icefish (*Chionodraco hamatus*, Lönnberg, 1905 [150]) [151,152,153], promoting the trafficking of particular genes on and off the sex chromosomes and playing a role in chromosomal rearrangement [154]. This phenomenon had been previously observed in the SDRs of the three-spine stickleback (*Gasterosteus aculeatus*, Linnaeus, 1758 [155]), which contains a Y-specific new unclassified repetitive element [41], and platyfish, in which a non-autonomous XIR Y-specific retrotransposon has been characterized [11,94].

A recent study compared TE landscapes on W chromosomes from high-quality avian genomes of six species spanning the avian tree of life from Paleognathae (emu with homomorphic sex chromosomes) and Neognathae (chicken, Anna’s hummingbird, kākāpō, paradise crow and zebra finch with heteromorphic sex chromosomes) [85]. The study revealed that the W chromosomes of birds are highly enriched in transcriptionally active TEs, including a majority of ERVs. The W chromosome likely acts as a sex-specific resource of retroelements, which may have numerous implications in emu (*Dromaius novaehollandiae*, Latham, 1790 [156]), Anna’s hummingbird (*Calypte anna*, Lesson, 1829 [157]), and paradise crow (*Lycocorax pyrrhopterus*, Bonaparte, 1850 [158]) [85]. Recent studies in reptiles have shown the presence of diverse Bov-B LINE groups in the snake lineage. Although males tended to show a higher copy number than females in some snake species, overall, no difference between sexes in Bov-B LINE copy number was observed [159]. Data are available on the abundance of L1 elements in snakes [160]. TRL1L, which shows a high sequence identity with the L1 nLTR retrotransposon family of the anole lizard (*Anolis carolinensis*, Voigt, 1832 [161]), has been investigated in several snakes, revealing truncated TRL1L insertions [162,163,164]. TRL1L shows a non-random distribution pattern on the W chromosomes of snakes, depending on the stage of W chromosome differentiation [164]. TRL1L sequences are present in higher copy numbers in the heterochromatic W chromosomes of the western whip snake (*Hierophis carbonarius*, Lacépède, 1789 [165]) and four-lined snake (*Elaphe quatuorlineata*, Lacépède, 1789 [166]) than in that of the Saharan sand viper (*Cerastes vipera*, Linnaeus, 1758 [167]), whose W chromosome is mostly euchromatic and homomorphic compared to the Z chromosome [163,167,168]. The preferential accumulation of L1s on the nearly completely heterochromatic W chromosome may be tolerated because of the effect of neutral selection and may contribute to the differentiation of heteromorphic sex chromosomes [83,169,170].

In contrast to snakes, L1s are abundant components (6%) in mammalian genomes [171], but are unable to transpose [172,173,174]. The relatively low copy number of L1s, with few full-length (active) copies, may be the result of a “purifying selection” that is very relaxed in snakes [40,175,176,177]. A recent study demonstrated that full-length L1s are subject to purifying selection in the human genome, while truncated L1s are essentially neutral [178]. Interestingly, non-functional L1s are predominantly located on the sex chromosomes in rodents of the genus *Microtus*, with distinct asymmetric distribution patterns on the Y and X chromosomes of different species. L1s are consistently located on the Y heterochromatin and are less abundant on the X euchromatin [169]. These distinct distribution patterns are consistent with the different processes. The presence of L1s on mammalian X chromosomes has been hypothesized to have functional significance given their involvement in X inactivation [179]. The low frequency of L1 interruptions in strata with high numbers of inactivated genes suggests selection against L1 interruptions in these regions. Individuals with interrupted L1s near inactivated genes were removed from the population more efficiently than individuals carrying interrupted L1s on the autosomes, in whom such interruptions are likely to be neutral [164]. Collectively, the results of TRL1L distributions on heterochromatic W chromosomes in snakes and on X chromosomes in mammals suggest a convergent trend between the two lineages concerning the differentiation processes of heteromorphic sex chromosomes. However, the reasons for the preferential TE accumulation in different lineages remain unclear.

## 4. Do Ty3/Gypsy TEs Facilitate Sex Chromosome Differentiation?

Substantial evidence suggests a close relationship between retroviruses and certain LTR retrotransposons, such as Ty1/Copia and Ty3/Gypsy [61,81,180,181,182,183]. Ty3/Gypsy TEs are phylogenetically more closely related to vertebrate retroviruses (Retroviridae) than to the Ty1/Copia class (Pseudoviridae) [184]. A few Ty3/Gypsy elements have a third open reading frame, putatively encoding an envelope protein, such as in the fruit fly (*D. melanogaster*) [181]. Thus, some Ty3/Gypsy-related elements display similarity to retroviruses in that they have an envelope (*env*) gene, which may indicate their infectious nature [185,186,187]. Several Ty3/Gypsy elements have occasionally been transmitted horizontally. For example, the envelope-encoding Gypsy element has jumped between species of the *D. melanogaster* subgroup [188], while the sea urchin retroviral-like SURL elements (no apparent envelope gene) have been transferred between echinoid species [189,190]. The Sushi retrotransposon, which belongs to the Ty3/Gypsy class, was originally observed in the Japanese pufferfish (*Fugu rubripes* Temminck and Schlegel, 1850 [191]) [192]. Other partial elements (mostly partial *pol* sequences) have been identified in lampreys, fishes, amphibians and reptiles [193,194,195,196]. The Hsr1 element in terrestrial salamanders (*Hydromantes* spp.) shows a high degree of similarity to the pufferfish Sushi element [191,195]. This suggests that Ty3/Gypsy elements are widely distributed across living organisms [196,197]. However, they are present in extremely low copy numbers in mammalian genomes [94].

Ty3/Gypsy elements are stable in nearly all fruit fly (*D. melanogaster*) strains studied to date, and their copies are generally located in centromeric and/or pericentromeric regions, as also observed in *D. virilis and D. subobscura* [198]. The papaya HSY region occupies only 13% of the Y chromosome [61]; however, analysis of HSY bacterial artificial chromosomes revealed that papaya HSY has a high repeat content [61]. Comparative genomics revealed that Ty3/Gypsy elements are highly accumulated in the SDR and account for 46.3% of the HSY and 37.7% of the corresponding X chromosome. By contrast, Ty1/Copia elements are less abundant than Ty3/Gypsy in both the HSY and the corresponding X chromosome. The Ty1/Copia content in the corresponding X chromosome is 1.3% higher than that in the HSY, suggesting that Ty1/Copia elements are not a major contributor to repeat accumulation in both the HSY and the corresponding X chromosome. This differs from the TE accumulation scenario in the broadleaf arrowhead (*Sagittaria latifolia*, Willd, 1805 [199]) Y chromosome, where Ty1/Copia elements are more abundant than Ty3/Gypsy elements [200].

In animal lineages, genome-wide SNP analyses of fishes and squamate reptiles have shown that most sex-specific or sex-linked loci are more strongly associated with Ty3/Gypsy than with other TEs [72,73,74,75,76,77,78]. This might be because of the role of Ty3/Gypsy elements in sex chromosome differentiation or the novel genomic impacts. It is also possible that partial Ty3/Gypsy accumulation on sex-specific or sex-linked loci follows a stochastic pattern within a restricted set of species, representing random homologies, given that only small sets of genomic regions in a restricted set of species are involved. Investigating the possible existence of Ty3/Gypsy elements in other organisms using fluorescence in situ hybridization mapping and whole-genome sequencing may further substantiate this hypothesis. The cumulative load of functional TEs from different classes on W/Y or other chromosomes has not yet been investigated in detail in many non-model species because of difficulties in the sequencing of W/Y chromosomes with highly repetitive sequences [3,45,48,144] and in the assignment of functional TE transcripts to particular loci [201]. Given these limitations, we expect that the transposition rates of particular TE families may have been underestimated and/or artificially standardized over evolutionary time [35,202].

## 5. Determination of the Crucial Role of Ty3/Gypsy in Sex Chromosome Evolution

Owing to the recent revolution in omics technologies, TE biology is one of the fastest evolving genomic research areas. However, our understanding of TE biology in terms of genome structure, activities, functions and effects remains limited. The assembly of heteromorphic sex chromosomes is challenging because of the high abundance of repeats. *De novo* assembly using long-read genome assemblers, such as PACBIO, and accurate annotation have enabled comparative genomic analyses across multiple species and different populations of the same species [203]. Recent advancements in assemblies have recovered missing repeats and resolved gaps in regions spanning mainly satellite arrays and sequences with TEs on sex chromosomes. The combination of chromosomics and chromosome-scale genomes with assembled sex chromosomes and advanced bioinformatic tools provide excellent resources to study sex-linked sequences for TE annotation and to evaluate their impact on sex chromosome differentiation and evolution [17,18,19,20,21,44,90,204,205]

Updated versions of assemblies for many model and non-model species have successfully identified male- and female-specific regions, such as the small 250 kb large male-specific region on the Y chromosome of the medaka [91] and the Z and W chromosomes of the half-smooth tongue sole [97]. Furthermore, bacterial artificial chromosome sequences of the Y-linked SDRs of the three-spined stickleback [41], southern platyfish [204] and salmonids [110] have been sequenced, but not yet fully assembled. Sex-specific or sex-linked TEs from assembled sex chromosome sequences can be targeted and comparatively analyzed. Candidate Ty3/Gypsy elements can be investigated to determine whether sex chromosomes preferentially accumulate this TE.

**Figure 2:**
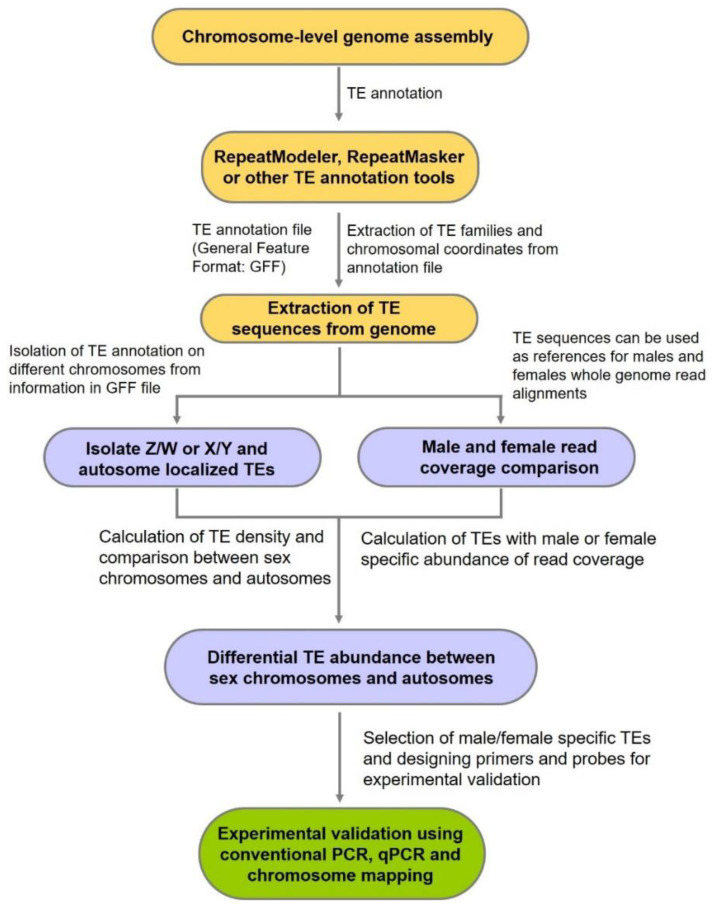
Workflow diagram on how to perform TE analysis to identify sex-differentiated regions harboring abundant Ty3/Gypsy elements and other elements important for sex chromosome differentiation.

Our previous studies revealed that in fish and reptilian species, Ty3/Gypsy elements share strong sequence similarity with sex-specific or sex-linked loci [72,73,74,75,76,77,78]. Thus, we hypothesize that this element contributed to the structural changes of the sex chromosomes (Figure 1). This hypothesis can be tested by comparing fully assembled XY or ZW sex chromosomes and annotating species-specific and sex-specific or sex-linked TE sequences. First, the assembled sex chromosomes should be investigated for repetitive elements using a set of annotation tools, such as RepeatModeler2, RepeatMasker, Extensive de novo TE Annotator (EDTA), RECON, RepeatScout and LTRharvest, and manually curated for sequences labeled as “LTR” or “unknown” [206,207,208,209,210,211]. Based on their underlying detection method, the annotation toolkits can be classified into three types: homology-based, structure-based and based on de novo identification of novel repetitive sequences [210]. Using custom python/bash scripts, Ty3/Gypsy sequences can be extracted from output gff3 files for sex chromosomes. A comparative analysis between Z and W or X and Y can then be performed to compute differential abundance and quantify variant copy numbers through different statistical approaches in R [212] (Figure 2). To further investigate the sex-specific abundance of Ty3/Gypsy, autosomes and sex chromosomes can be compared. Identifying sex-specific or sex-linked elements on the basis of mapped read coverage and calculating the ratio of male/female sequence reads mapped with Ty3/Gypsy can provide insights into copy number variations and sex-linked amplification. Transcriptome data of male and female gonads can be explored to estimate Ty3/Gypsy differential expression [213,214].

Further, it is essential to compare Ty3/Gypsy elements within the recombining and non-recombining regions between Z and W or X and Y to understand whether these elements drive sex chromosome differentiation. To evaluate the state of recombination, the sequence similarity of X or Z with Y or W, respectively, and with autosomes can be determined. This may result in the detection of homologous regions between both sex chromosomes (also known as pseudoautosomal regions). Y- or W-linked unique regions, representing SDRs, may also be identified. Pseudoautosomal regions and SDRs can be further investigated for the differential occurrence of Ty3/Gypsy using the techniques described above.

Classical methods should be employed to validate the computational analysis results. These methods include PCR metabarcoding to analyze the specificity and diversity of TE elements, chromosome fluorescence in situ hybridization mapping to determine chromosome localization, and qPCR to comparatively quantify TE copy numbers in male and female genomes. The microarray and RNASeq data available in the database could also be utilized to assess the expression analysis of genes and TEs associated with SD genes. Eventually, multiple approaches can be integrated to identify sex-specific or sex-linked TE loci. Analysis of Ty3/Gypsy interruptions can provide insights on selective constraints of Ty3/Gypsy elements. Insertion into Ty3/Gypsy sequences that are beneficial for the host would result in their disruption and loss of function. Individuals carrying such “knock-out” Ty3/Gypsy elements would undergo negative selection and may or may not disappear from the population. Thus, analyzing Ty3/Gypsy interruptions offers a novel means of investigating selective pressures on TEs in genomes.

TE analysis can provide exciting insights into how selective constraints of genomic loci harboring these elements reshape sex chromosomal evolution. Identifying the real targets of selection, including TEs, is challenging, but allows estimating whether any positively selected regions spanning TEs drive sex chromosome evolution. How and why sex chromosomes gain TEs is not fully understood. Studies have reported that these elements contribute to genome novelties and mediate chromosomal rearrangements and thus are expected to be involved in sex chromosome differentiation. Studying young sex chromosomes from fishes, amphibians, reptiles and plants rather than old and heteromorphic sex chromosomes from mammals and birds that are at advanced states of differentiation may help clarify the process of TE accumulation.

## 6. Final Considerations

Sex is an essential aspect for understanding the biology of any organism. Genome-level studies in many model and non-model species have elucidated the genetic mechanisms associated with sex chromosome differentiation. We highlight several facets of TEs in relation to sex that affect each other as co-evolutionary phenomena. TEs are ubiquitous genome components, but differ widely in their abundance. The vast majority of TEs have deleterious effects on the host genome because they promote gene mutations and chromosomal rearrangements, and are usually efficiently eliminated by selection. The non-random distribution of TEs on sex chromosomes suggests that, in addition to several sex-specific or sex-linked genes, sex-specific or sex-linked loci encoding certain types of TEs, such as Ty3/Gypsy, may drive sex chromosome differentiation. The linkage of TE insertions with a locus under strong positive selection would generate the conditions required for this mechanism. By contrast, the restriction of W/Y chromosome recombination may create hundreds of linked genes. Purifying selection, acting against deleterious mutations, may be greatly reduced [3]. Each TE copy has a probability of fixation that depends on the selective pressure, host population size and recombination frequencies in the surrounding genomic DNA. For functional TEs located on the W/Y chromosome, the net selection coefficient for deleterious mutations should grow with functional TE accumulation up to the point where most W/Y chromosomes have a detrimental effect on fitness [39]. We postulate that, when studying the evolution of non-recombining chromosomes, one should always consider the potential impact of TEs on fitness. This review considers evidence reporting the impact of TE accumulation on sex chromosomes. Our hypothesis of how TE accumulation impacted SD genes in different biological functions related to sex determination should be further investigated. SNP-based studies can also be integrated with transcriptomics and epigenomic level analyses to reveal the candidate SD genes regulated by accumulated TEs in the SDR to promote a better understanding. This may allow a better understanding of W/Y chromosome evolution and function.

## Data Availability

Not applicable.
